# Rapid identification of fruit length loci in cucumber (*Cucumis sativus* L.) using next-generation sequencing (NGS)-based QTL analysis

**DOI:** 10.1038/srep27496

**Published:** 2016-06-07

**Authors:** Qing-zhen Wei, Wen-yuan Fu, Yun-zhu Wang, Xiao-dong Qin, Jing Wang, Ji Li, Qun-feng Lou, Jin-feng Chen

**Affiliations:** 1 State Key Laboratory of Crop Genetics and Germplasm Enhancement, College of Horticulture, Nanjing Agricultural University, Nanjing 210095, China

## Abstract

The cucumber (*Cucumis sativus* L.) exhibits extensive variations in fruit size and shape. Fruit length is an important agronomic and domesticated trait controlled by quantitative trait loci (QTLs). Nonetheless, the underlying molecular and genetic mechanisms that determine cucumber fruit length remain unclear. QTL-seq is an efficient strategy for QTL identification that takes advantage of bulked-segregant analysis (BSA) and next-generation sequencing (NGS). In the present study, we conducted QTL mapping and QTL-seq of cucumber fruit length. QTL mapping identified 8 QTLs for immature and mature fruit length. A major-effect QTL *fl3.2*, which explained a maximum of 38.87% of the phenotypic variation, was detected. A genome-wide comparison of SNP profiles between two DNA bulks identified 6 QTLs for ovary length. QTLs *ovl3.1* and *ovl3.2* both had major effects on ovary length with a △ (SNP-index) of 0.80 (P < 0.01) and 0.74 (P < 0.01), respectively. Quantitative RT-PCR of fruit size-related homologous genes localized in the consensus QTL *FL3.2* was conducted. Four candidate genes exhibited increased expression levels in long fruit genotypes. Our results demonstrated the power of the QTL-seq method in rapid QTL detection and provided reliable QTL regions for fine mapping of fruit length-related loci and for identifying candidate genes.

Cucumber, *Cucumis sativus* L. (2n = 2x = 14), originates from the southern Himalayas and has been cultivated in India for at least 3000 years. Cucumber fruits exhibit extensive variation in shape or size between and within cultivated varieties resulting from long-term domestication. Recent studies categorized cucumbers into 4 geographic groups and 6 market classes based on various phenotypes and consuming properties, with fruit length being one of the most prominent traits^1^,^2^. For example, wild cucumbers typically bear small spheroid fruits ~4 cm in length. In contrast, North China fresh market cucumber fruits are at least 25 cm in length, and the European greenhouse cucumber is an intermediate type at 10 to 15 cm^2^,^3^.

Numerous studies have been performed to demonstrate the genetic and molecular basis controlling cucumber fruit length. It is now widely accepted that fruit length is determined by quantitative trait loci (QTLs) and easily influenced by cultivation conditions and environment. The first QTL mapping for cucumber fruit length was reported by Kennard and Havey, who used F_3_ populations from a cross between GY14 and P1432860^4^. With an F_2_ population and 224 RILs derived from a narrow cross of S94 (North China type) and S06 (European greenhouse type), Yuan *et al.*^5^,^6^ identified 6 and 7 QTLs for immature fruit length (FL), respectively. Miao *et al.*^7^ mapped 6 QTLs for FL and MFL (mature fruit length) on LG1, LG5 and LG6, with 5 QTLs identified in both growing seasons. Nevertheless, a previous study found that QTLs for fruit shape (e.g., fruit length and diameter) were less consistent across years and spacing than earliness and yield components^8^. Resequencing of 115 cucumber lines generated a genomic variation map that characterized 5 QTLs for fruit length, and *Csa3G199660* localized within QTL *fl3.1* was considered as a candidate gene^1^. More recently, we constructed a SNP (single-nucleotide polymorphism)-based saturated genetic map using specific-length amplified fragment (SLAF) sequencing and identified 6 QTLs for FL and MFL with an F_2_ population^9^. Using RIL populations developed from North China type × semi-wild cucumber inbred lines, Bo *et al.*^10^ detected 5 QTLs for MFL (*fl1.1, fl3.1, fl4.1, fl6.1*, and *fl7.1*). Weng *et al.*^2^ performed QTL analysis with three QTL models and multiple populations, detecting 29 consistent and distinct QTLs for fruit size/shape in different developing stages. Analysis of the available fruit size QTLs resulted in 12 consensus QTLs and a dynamic view of genetic control of cucumber fruit development. However, given the complex genetic basis of quantitative traits plus influences caused by different mapping population and environments, additional efforts are still needed to screen for reliable QTL regions and identify candidate genes.

Cucumber fruits generally develop from an enlarged inferior ovary followed by increases in cell division and cell expansion after fertilization or fruit set before ripening. The process of fruit elongation begins almost immediately after pollination and is typically completed in 12 to 16 days^11^. To understand the molecular events during early fruit development, transcriptome profiling was conducted with different cucumber lines, associating cucumber kinesin genes as well as microtubule and cell cycle related genes with fruit size/development regulation^3^,^12^,^13^. Compared with the knowledge of molecular biology of early fruit development in cucumber, insights into the genetic basis were very limited. Weng *et al.*^2^ conducted QTL mapping of ovary length (OvL), diameter (OvD) and ovary number (OvN) as well as the relationships among OvL, FL and MFL. The results indicate that factors regulating fruit length are largely determined pre-anthesis and that OvL is a good predictor for FL and MFL. Three QTLs for OvL and four QTLs for OvD were mapped on all seven cucumber chromosomes, with one QTL for each chromosome. Nevertheless, the three QTLs for OvL together could only explain 28.8 to 38.0% of phenotypic variations; thus, it is possible that additional QTLs not detected exist in the RIL population.

Traditional QTL mapping typically requires a segregating population between two parents showing contrast phenotypes of target traits and the selection of polymorphic DNA markers for linkage analysis. The process of polymorphic marker screening and genotyping is often labour-intensive and time-consuming^14^. QTL-seq is proposed as an efficient strategy for rapid identification of QTLs, which takes advantage of bulked-segregant analysis and high-throughput genotyping using next-generation sequencing (NGS). This approach has been applied to detecting QTLs in rice, cucumber, tomato, and chickpea^15^,^16^,^17^,^18^,^19^. Previously we constructed a high-density cucumber genetic map compromising 1800 SNPs using F_2_ populations derived from CC3 × NC76. In this study, we examined the correlation coefficient between FL and MFL and between OvL and MFL with F_3_ family and RIL populations from the same cross. Traditional QTL mapping and QTL-seq were performed at different development stages over three years. A total of 14 QTLs were detected for fruit length on five cucumber chromosomes. Integration of QTL information from the present study allowed us to identify consensus QTLs for fruit length in the cucumber. Genome-wide identification of fruit size homologous genes was performed using 74 fruit size-related sequences in melon. The expression levels of candidate genes localized in a consensus QTL region on chromosome 3 were analysed by quantitative RT-PCR.

## Results

### Phenotypic evaluations of fruit length in CC3 × NC76 populations

**Figure 1:**
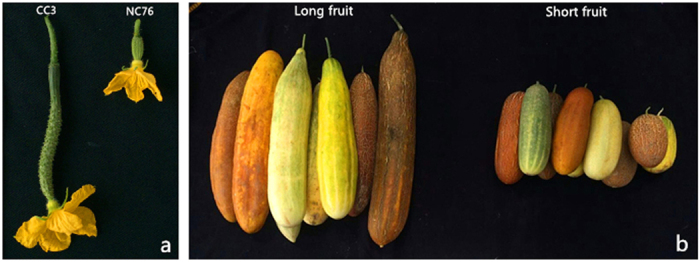
Fruits of CC3, NC76 and RIL populations. (**a**) Ovaries of the maternal CC3 (left) and paternal NC76 (right) on day-of-anthesis; (**b**) Extreme long and short mature fruits harvested from RIL populations.

In the present study, two cucumber inbred lines, ‘CC3’ and ‘NC76’ (Fig. 1a), were crossed to develop segregating populations for QTL analysis of fruit length. Phenotypic data of FL and MFL were collected using F_3_ families in spring 2013 and autumn 2014, and OvL and MFL data of F_7_ RIL populations (Fig. 1b) were collected in spring 2015. Detailed phenotypic data, frequency distribution among tested materials, and results from statistical analysis of fruit length are presented in Supplementary Tables S2 and S3.

**Figure 2:**
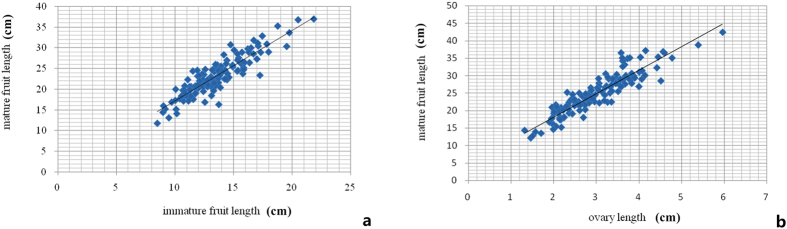
Correlation coefficiency among immature fruit length, mature fruit length and ovary length. (**a**) Correlation coefficiency between immature length and mature fruit length using the means of F_3_ families in spring 2013 and autumn 2014. The X-axis represents immature fruit, and the Y-axis represents mature fruit length; (**b**) Correlation coefficiency between mature fruit length and ovary length using F_7_ RILs in spring 2015. The X-axis refers to ovary length on day-of-anthesis, and the Y-axis refers to mature fruit length.

We calculated correlation coefficients among OvL, FL and MFL from two segregating populations in three growing environments. The correlation coefficients between FL and MFL in spring 2013 and autumn 2014 were 0.852 and 0.813, respectively (Supplementary Table S3). Different environmental conditions were likely the main factors leading to differences in correlation from two seasons. The correlation coefficient was calculated to be 0.896 using the average fruit length data of the two seasons (Fig. 2a), suggesting that fruit length at immature stage is significantly correlated with that at mature stage. Considering ‘immature’ as a dynamic period and given that fruit length is constantly changing, ovaries and mature fruits are in a relatively stable stage at the start and end of cucumber fruit development process, thus generating more effective phenotypic data. Thereby, correlation coefficients between OvL and MFL in the same RIL populations in spring 2015 were calculated. We observed a significant correlation between OvL and MFL, with a coefficient value of 0.917 (Fig. 2b). In the melon, the ovary and mature fruit morphology also have a high correlation, and fruit shape is predominantly determined pre-anthesis^20^,^21^. Phenotypic analysis in the present study suggests that cucumber early fruit development has a direct impact on final fruit length and that common QTL regions play important roles in determining cucumber fruit length, despite the chaos caused by differences in growing environments, nutrient conditions and pollination quality.

### QTL-mapping

We previously constructed a SNP-based genetic map by resequencing two parents (CC3 and NC76) and F_2_ populations using SLAF-seq^9^. To correct genotyping errors from NGS data, Highmap^22^ was employed to re-genotype the SNPs in this study. Although all the 1800 SNPs were preserved, the positions and orders of some SNPs on linkage map were corrected, generating a genetic map with better synteny when aligned to 9930 reference genome (Supplementary Fig. S1). QTL analysis of FL and MFL was performed using R/qtl (http://www.rqtl.org/) and MapQTL®6 (https://www.kyazma.nl/index.php/mc.MapQTL/) software packages to produce consensus QTL regions.

In total, eight QTLs were identified for FL and MFL in four cucumber chromosomes (chromosomes 1, 3, 4 and 6). Information for QTLs detected in QTL mapping is provided in Table 1. Figures for all QTLs with LOD sores were presented in Supplementary Fig. S2. Three QTLs, *mfl1.1*, *mfl3.2*, and *fl3.2* were detected using two software packages in both growing seasons with slightly different physical intervals of each QTL locus. For QTL *fl3.2*, despite the slight shift of start and end locations detected with MapQTL in two seasons, the high phenotypic variation explained by this locus (>36%) and the same chromosome regions identified using R/qtl in both seasons suggest that this locus has a major effect on cucumber immature fruit length. There were four minor-effect QTLs (*fl1.1*, *fl3.1*, *mfl3.1*, and *fl4.1*) detected exclusively with R/qtl in either spring 2013 or autumn 2014, in which three QTLs for FL were noted. QTL *fl6.1* was detected with two software packages in both seasons except for R/qtl in spring 2013. The chromosome interval of QTL *fl3.2* (22.71–28.20 Mb) overlapped with that of *mfl3.2* (25.37–30.54 Mb) in a 2.83 Mb region, explaining at least ~30.87% of fruit length variation. Thus, *fl3.2* and *mfl3.2* might simultaneously control elongation of immature and mature fruit. QTLs located in close chromosome regions were considered as a consensus QTL interval, and the maximum span of flanking markers of the QTL locus was obtained. For example, QTL *mfl1.1* on chromosome 1 spanned a physical interval from 13.30 to 17.66 Mb.

### QTL-seq

**Figure 3:**
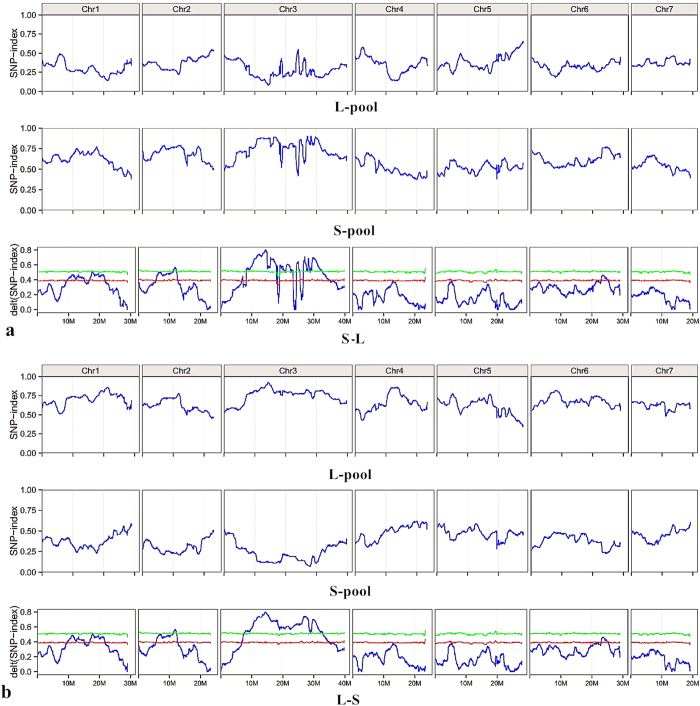
SNP-index graphs of L-pool, S-pool and Δ (SNP-index) graphs from QTL-seq analysis. The Δ (SNP-index) plot with statistical confidence intervals under the null hypothesis of no QTL (green, P < 0.01; red, P < 0.05). The *X*-axis represents the position of seven chromosomes, and the *Y*-axis represents the SNP-index. The SNP-index was calculated based on a 1 Mb interval with a 10 kb sliding window. a: using CC3 genome sequence as the reference genome; b: using NC76 genome sequence as the reference genome.

Illumina high-throughput sequencing generated 13,324,160,800 and 10,859,863,600 short reads (100 bp in length) from the L-pool and the S-pool, with a coverage of 91.34% (42.69-fold genome coverage) and 92.28% (34.22-fold genome coverage), respectively. The minimum Q20% was 93.84% of the S-pool, and the maximum was 94.26% of the L-pool. The lowest sample coverage was 91.34% of the L-pool. The effective sequencing depths for CC3 and NC76 were 16.41-fold and 21.04-fold genome coverage, respectively, which guarantees the accuracy of subsequent analysis. The results from QTL-seq are presented in Table 2. Sequence data were trimmed and filtered prior to analysis. Two paternal lines, CC3 and NC76, in the present study were resequenced because no reference genome sequences were available for these two cucumber lines. First, short reads from both parents were aligned to the 9930 genome to obtain two consensus sequences, which were used as reference sequences in the subsequent analysis. Second, reads obtained from two DNA-bulks (L-pool and S-pool) were aligned to consensus sequences to identify single nucleotide polymorphisms (SNPs). To identify genomic regions associated with fruit length, we evaluated the proportion of SNP bases available between L-bulk and S-bulk. The SNP-index was calculated for each SNP identified in the genome, and the average SNP-index within a 1 Mb window size was computed using a 10 kb step increment. SNP-index graphs for L-pool and S-pool were plotted by aligning an average SNP-index against the position of each sliding window in the CC3 reference genome (Fig. 3a). The Δ (SNP-index) was calculated and plotted using the information from two graphs for L-bulk and S-bulk (Fig. 3a). Δ (SNP-index) = 1 means that the bulked DNA exclusively comprises the NC76 genome (S–L). To ensure the accuracy of results, graphs of SNP-index and Δ SNP-index using the genome sequence of NC76 as a reference genome were also generated (Fig. 3b), whereby Δ (SNP-index) = 1 means that the bulked DNA exclusively comprises the CC3 genome (L–S).

DNA sequences of L-bulk and S-bulk were expected to be identical except for regions harbouring QTLs relevant to ovary length where unequal contributions from CC3 and NC76 paternal genomes may exist. In total, we identified six QTLs underlying ovary length in RIL populations (*ovl1.1*, *ovl1.2*, *ovl2.1*, *ovl3.1*, *ovl3.2*, and *ovl6.1*) across four cucumber chromosomes (Table 3). Four QTLs were detected using both parent genome sequences, whereas two QTLs were only detected with the NC76 reference genome. In addition, sequence alignment using different paternal reference genomes may result in different chromosome locations. Statistical confidence intervals of Δ (SNP-index) were calculated for all the SNP positions with given read depths under the null hypothesis of no QTL (see Materials and Methods). The chance that Δ (SNP-index) becomes higher than 0.52 as observed for the chromosomal region of 10.73–12.76 Mb is P < 0.01 under the null hypothesis. Likewise, the chance that Δ (SNP-index) becomes higher than 0.48 as observed for the chromosomal region of 16.95–20.20 Mb is 0.01 < P < 0.05 under the null hypothesis. The six Δ (SNP-index) peaks for OvL were statistically significant (the peaks for *ovl2.1*, *ovl3.1* and *ovl3.2*, P < 0.01; and the peaks for *ovl1.1*, *ovl1.2* and *ovl6.1*, 0.01 < P < 0.05). QTL *ovl3.2*, spanning a physical interval of 25.97 to 30.55 Mb on chromosome 3, was a major-effect QTL with a Δ (SNP-index) of 0.74 (P < 0.01). Another QTL that had a major effect on the ovary length was *ovl3.1* ranged from 11.12 Mb to 16.45 Mb with a peak ΔSNP-index value of 0.80 (P < 0.01).

### Comparison of QTL detection using two NGS-based methods

**Figure 4:**
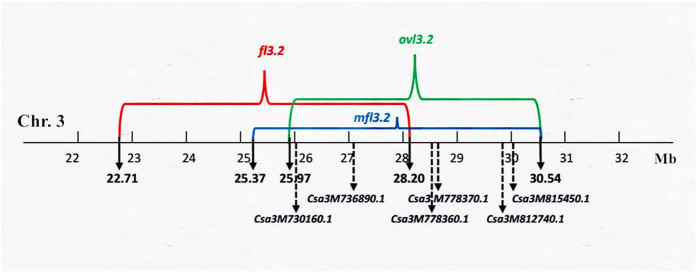
The co-localization of six fruit size homologues within chromosomal interval of the consensus QTL *FL3.2*. Chromosomal intervals of *ovl3.2* (green), *fl3.2* (red) and *mfl3.2* (blue) are given in Mb.

The two strategies employed in the present study take advantage of high-throughput NGS. QTL-seq of 2015 RIL trails identified 6 QTLs for ovary length (*ovl1.1, ovl1.2, ovl2.1, ovl3.1, ovl3.2*, and *ovl6.1*). QTL mapping of 2013 and 2014 F_3_ families using a revised saturated SNP-based genetic map detected 3 QTLs for mature length (*mfl1.1, mfl3.1* and *mfl3.2*). RIL trails are more suitable for detecting minor-effect QTLs, which may partly explain why more QTLs were identified when using RILs. Two overlapping regions were noted between chromosomal intervals of *mfl1.1* (13.30–17.66 Mb) and *ovl1.2* (16.92–20.20 Mb) as well as *mfl3.2* (25.37–30.54 Mb) and *ovl3.2* (25.97–30.55 Mb). Although the QTL interval of *ovl1.1* (10.42–12.09 Mb) did not overlap with *mfl1.1*, it was found to be located between *fl1.1* (7.27–9.18 Mb) and *mfl1.1*, where a dozen QTLs were detected for fruit length and defined as *FS1.1* and *FS1.2* by Weng *et al.*^2^. Given that the genomic regions of *fl3.2* (22.71–28.20 Mb)*, mfl3.2* and *ovl3.2* that control fruit length at different development stages overlapped (as shown in Fig. 4), we defined QTL *FL3.2* as a consensus QTL that potentially played a consistent role in cucumber fruit elongation through all growing phases. The maximum interval was obtained for *FL3.2* ranging from 22.71 to 30.55 Mb (7.84 Mb), whereas the actual overlapping region spanned 2.33 Mb (25.97–28.20 Mb). However, differences in QTL detection were present in several cases. The QTL interval of *ovl3.1* did not overlap with *mfl3.1*; QTLs *ovl2.1* and *ovl6.1* were only detected for OvL. A high correlation coefficieny was observed between OvL and MFL using phenotypic data from 2015 RIL trails. Whereas QTL-seq of OvL was conducted with 2015 RILs, QTL mapping of MFL was conducted with 2013 and 2014 F_3_ families. Different mapping populations and QTL analysis methods possibly contributed to the differences in chromosomal locations of QTLs for OvL and MFL.

### Analysis of genome-wide homologous genes for fruit size

**Figure 5:**
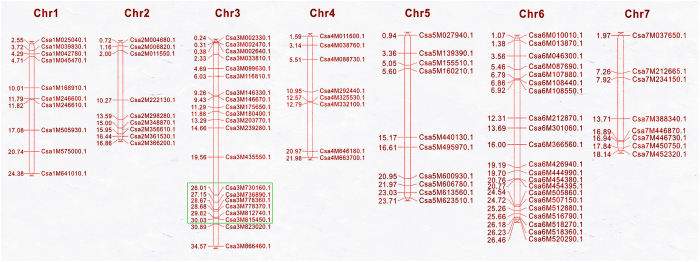
Distribution of homologous genes for fruit size detected on seven cucumber chromosomes. Physical locations for fruit size homologues on cucumber chromosomes (Chr. 1–7, on the top). Chromosomal distances (Mb) are on the left, and gene names are on the right. The six homologous genes co-localized within QTL *FL3.2* are depicted by green box.

**Figure 6:**
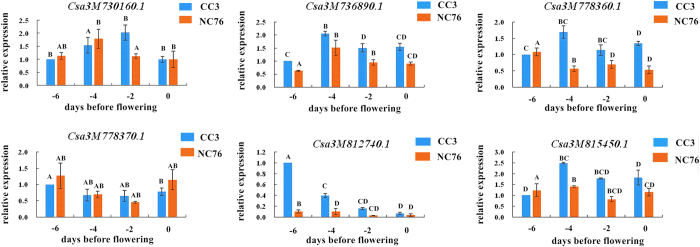
qRT-PCR results of six candidate genes localized in chromosome regions of the consensus QTL *FL3.2.* The blue bars represent CC3, and the red bars represent NC76; The *X*-axis indicates 0, 2, 4, 6 days before anthesis, whereas the *Y*-axis indicates expression levels. The alphabets (A–D) represent significant levels of gene expression.

A total of 74 homologues of fruit size-related genes were identified in the melon genome using tomato or *Arabidopsis thaliana* gene family sequences as queries^23^. With the hypothesis that gene families associated with fruit size may have similar roles in regulating fruit morphology, especially in phylogenetically close species^10^, we used the 74 melon fruit size-related sequences as queries to blast against the 9930 cucumber genome sequence^24^ (Version 2). Homologues of all 74 gene sequences were identified in the cucumber genome. Optimal alignment gene homologues and associated information of each homologous gene are presented in Supplementary Table S4. The distribution of homologous genes on cucumber chromosomes was shown in Fig. 5. To further understand the function of these homologs, gene ontology (GO) term enrichment analysis (P ≤ 0.05) was performed. The most enriched GO terms were ‘cellular process’ in biological process (GOBP) group (green in Fig. S3), ‘cell part’ in molecular function (GOMF) group (blue in Fig. S3) and ‘binding’ in cellular component (GOCC) (red in Fig. S3), respectively. Six genes (*Csa3M730160.1, Csa3M736890.1, Csa3M778360.1, Csa3M778370.1, Csa3M812740.1*, and *Csa3M815450.1*) from predicted fruit-size homologues co-localized within the chromosomal region of the consensus QTL *FL3.2*; thus, these genes are considered as possible candidate genes for fruit length. GO terms were assigned to three candidate genes (*Csa3M730160.1, Csa3M736890.1*, and *Csa3M812740.1*) (Supplementary Table S4). *Csa3M812740.1* was involved in multiple biological processes (GOBP) such as embryo morphogenesis, floral organ development and cell growth, etc. Gene annotation of the six fruit size homologues using Cucurbit genomics database (http://www.icugi.org) was shown in Supplementary Table S4. Furthermore, six primers pairs were designed based on cDNA sequences to perform qRT-PCR analysis. Expression levels of all candidate genes in ovaries of 0, 2, 4, and 6 days before flowering from both parents were measured. Six genes except for *Csa3M778370.1* and *Csa3M815450.1* exhibited increased expression levels in CC3 compared with NC76, especially on ovaries of 2 and 4 days before flowering, including *Csa3M730160.1* and *Csa3M736890.1* located in the 2.23Mb overlapping interval of *FL3.2* (Fig. 6). *Csa3M778370.1* exhibited no obvious regularity in expression patterns. *Csa3M812740.1* had significantly increased expression in the ovaries of CC3, corresponding to its involvement in multiple biological processes that determine the development of ovary such as embryo morphogenesis, floral organ development and cell growth, etc. Gene annotation suggested that *Csa3M730160.1* and *Csa3M778360.1* encoded members from ovate family protein (OFP); *Csa3M736890.1* encodes a member from IQ domain family. The functions of *Csa3M778370.1* and *Csa3M815450.1* are still unknown.

## Discussion

Early fruit development of many horticultural crops, including the cucumber, can be divided into three phases: development of the ovary, cell division, and cell expansion^25^. The increase in cucumber fruit size is often mirrored by the increase in cell number and size^26^. Insight into the genetic and molecular mechanisms of early fruit development is important to understand the factors determining fruit yield and quality in cucumber production. Cell division typically occurs approximately one week post-anthesis, whereas cell enlargement persists throughout the process of fruit development^27^. In the melon, the ovary and mature fruit morphologies exhibit a high correlation^20^,^21^, and fruit shape is predominantly determined pre-anthesis. In the present study, correlation analysis was conducted based on ovary, immature and mature fruit length in F_3_ families and RIL populations derived from CC3 × NC76 in 2013, 2014 and 2015. The correlation coefficient between FL and MFL was 0.896, whereas that of OvL and MFL was 0.917 in RILs. The high correlation coefficient, especially between OvL and MFL, suggests that early fruit development may directly determine final fruit length. These results were consistent with that of Weng *et al.*^2^, in which strong positive correlations were also observed among OvL, FL, MFL and OvN using F_2_, F_3_ families and RIL populations derived from 9930 × Gy14. Thus, dynamic changes in fruit length during fruit development are consistent, i.e., the fruit length of NC76 was consistently shorter than that of CC3 through all growing stages. OvL could serve as good indicator for FL and MFL in the QTL analysis of cucumber fruit length.

High-throughput NGS has revolutionized the approach to QTL analysis of complex traits in rapid marker discovery and map construction. We conducted rapid QTL detection of cucumber fruit length using two NGS-based strategies. QTL mapping requires a linkage map with abundant markers, whereas QTL-seq does not require the construction of segregation populations or screens for polymorphic markers. However, QTL-Seq can provide a rough location of a particular QTL, and additional QTL analysis is still needed to refine locations and narrow chromosomal intervals. Highmap^22^ was employed to re-genotype the SNPs from a pre-constructed genetic map using SLAF-seq^9^. A revised SNP-based genetic map (1800 SNPs) was generated with better synteny when aligned to 9930 reference genome (Fig. S1). QTL mapping was performed in F_3_ families in spring 2013 and autumn 2014 using the revised SNP genetic map. QTL-seq was performed using 2015 RIL populations. In total, 14 QTLs associated with OvL, FL and MFL were detected on all cucumber chromosomes except for chromosomes 5 and 7. Given the high correlation coefficiency between OvL and MFL from 2015 RIL trails, corresponding correlated chromosomal intervals of QTLs for OvL and MFL are expected. Indeed, two overlapping regions were identified between QTL intervals of *mfl1.1* and *ovl1.2*, and *mfl3.2* and *ovl3.2*. We defined a consensus QTL *FL3.2* for OvL, FL and MFL that harbors *ovl3.2, fl3.2* and *mfl3.2*, which was considered as a major-effect QTL for cucumber fruit length. This result is consistent with that of our previous study that identified two major QTLs (*fl3.2* and *mfl3.2*) for fruit length. However, there are several cases that QTL locations did not overlap such as *ovl3.1* and *mfl3.1*. One reasonable explanation is that different environment conditions, populations and QTL analysis methods affect the results. In addition, although OvL and MFL are highly correlated, they are considered as different traits.

A number of fruit length QTLs have been identified in previous studies and are distributed on all seven chromosomes^2^,^4^,^5^,^6^,^8^,^9^,^10^,^28^,^29^. Assuming that QTLs at the same or close chromosome locations belonged to the same QTL locus, those from different studies may share common genetic mechanisms underlying fruit elongation. Weng *et al.*^2^ identified 12 consensus QTLs for fruit size by the synthesis of information from 29 consistent and distinct fruit size QTLs. The four QTLs on chromosome 1 in the present study were mapped in the consensus QTL region of *FS1.1* and *FS1.2* for fruit size (Table 1). Similarly, QTL *ovl6.1* for ovary length localized in consensus QTL regions for *FS6.1* and *FS6.2* which harboured QTLs for FL identified by several studies^2^,^6^,^7^,^27^. The QTL for FL on chromosome 4 (*fl4.1*) was close to several QTLs detected by three previous studies^2^,^6^,^10^. QTL *fl6.1* has a highly consistent chromosome interval in different mapping populations and environmental conditions^9^. Although *ovl3.2* in the present study has a different chromosome location from that detected by Weng *et al.*^2^, both were located in consensus QTL *FS3.2*. We detected two QTLs for ovary length (*ovl1.1* and *ovl1.2*) in the RIL populations. One QTL (*qOvL1.1*) was detected by Weng *et al.*^2^, which was close to the chromosome location of *ovl1.1*. Previously, cucumber chromosome 2 has only one QTL identified for immature fruit length, whereas we detected a QTL for ovary length (*ovl1.2*) located within chromosome intervals of *FS2.1*. QTLs *fl3.1* and *mfl3.1* were localized in the QTL region of *FS3.1*, whereas *ovl3.1* was close to a QTL for immature fruit length (*fl3.1)* identified by Wang *et al.*^29^. Many factors could influence results of QTL mapping from different studies for the same trait, such as different mapping populations (F_2_, F_3_ and RIL), the season, different growth stages and environment conditions. However, a more reasonable explanation is that cucumber fruit size has undergone domestication or diversifying selection for different taxonomic groups or specialized market classes. Thus, the underlying genes/QTLs for the same trait may be differentially expressed in cucumber lines of different market classes.

The molecular and genetic mechanisms underlying fruit development in the tomato (*Solanum lycopersicum*) have been extensively studied, with several genes/QTLs for fruit size or shape being fine-mapped and cloned^30^,^31^,^32^. Four genes regulating fruit shape (*SUN* and *OVATE*) and weight (*CNR/FW2.2* and *SlKLUH*/*FW3.2*) are associated with fruit elongation. *SUN* encodes a protein that belongs to the IQ domain family; *OVATE* encodes a protein in the ovate family protein (OFP). *CNR/FW2.2* encodes one of the cell number regulators (CNR). Putative orthologues of genes regulating tomato fruit morphology have been proposed as candidate genes in other species such as pepper and cherry^33^,^34^,^35^. With the assumption that fruit morphology in different taxa could be controlled by genes from certain ancestral gene families, Monforte *et al.*^23^ identified 74 homologues of the CNR, CYP78A, OFP, SUN, WOX, and YABBY gene families for fruit shape and size in the melon genome. We used the 74 melon gene sequences as queries to BLAST against the 9930 cucumber genome sequence^24^ (Version 2). Homologues of all 74 gene sequences were identified in the cucumber genome, and this finding is consistent with results reported by Bo *et al.*^10^. We employed a different E value and retained all the 87 genes detected; the blast frequency was calculated for each gene as another reference besides identity. The consensus QTL *FL3.2* compromised six homologous genes for fruit size, whereas GO terms were assigned to three of them (*Csa3M730160.1, Csa3M736890.1*, and *Csa3M812740.1*) (Supplementary Table S4). Notably, all six predicted genes co-localized in the QTL region detected for *ovl3.2*. Expression levels of six genes except for *Csa3M778370.1* and *Csa3M815450.1* were higher in CC3 compared to NC76, especially 2 and 4 days before flowering (Fig. 6). *Csa3M812740.1* had significantly increased expression in the ovaries of CC3, which corresponded to its involvement in multiple biological processes such as embryo morphogenesis, floral organ development and cell growth, etc. Gene annotation using Cucurbit genomics database (http://www.icugi.org) suggests that *Csa3M730160.1* and *Csa3M778360.1* encode members from OFP family; *Csa3M736890.1* encodes a member from IQ-domain family. The functions of *Csa3M778370.1* and *Csa3M815450.1* are lacking. The increased levels that exhibited by the four cucumber homologous genes in CC3 and corresponding functions implicate the putative involvement in regulating cucumber fruit length. However, additional work is still needed to validate the roles of homologous genes in determining cucumber fruit size. Seven kinesin genes (*CsKF1* to *CsKF7*) in the cucumber genome were associated with rapid cell production and cell expansion via transcriptome analysis of cucumber lines with different fruit sizes^13^. Weng *et al.*^2^ anchored 74 fruit size homologous genes and the seven kinesin genes onto a cucumber SNP map, most of which are located within the QTL regions detected from the present and previous studies. Nevertheless, one QTL locus often harbours multiple gene homologs. Genomic intervals of QTL loci still need to be narrowed down to determine candidate genes for cucumber fruit length.The results from the present study demonstrate the power of combined QTL mapping and QTL-seq method in multiple traits and provide insights into the genetic mechanisms underlying the cucumber fruit length.

## Materials and Methods

### Plant materials and phenotypic data collection

Two cucumber inbred lines, ‘CC3’ and ‘NC76’, were used to develop segregating populations for QTL analysis in this study (Fig. 1a). CC3, the maternal line, is a typical North China fresh market type with slim immature fruits and a length of 20~30 cm at commercial harvest stage. NC76, the paternal line, is a US slicing cucumber that bears short immature fruits of 7 to 10 cm in length at commercial harvest stage. In this study, a single F_1_ plant from the cross between CC3 and NC76 was self-pollinated to produce 148 F_2_, and 133 F_2_-derived F_3_ families were used in QTL mapping. In total, 135 F_7_ recombinant inbred lines (RILs) developed through single seed descent were used for QTL-seq (Fig. 1b).

Two parental lines (CC3 and NC76), F_3_ families and F_7_ RILs were grown in a greenhouse at Jiangpu Cucumber Research Station of Nanjing Agricultural University (JCRSNAU), Nanjing, China. The soil media was 25% peat + 25% cinder + 50% perlite. For the evaluations of immature and mature fruit length (FL and MFL), six plants randomly selected from F_3_ families were grown in July 2014, and three plants from each F_7_ RILs were planted to measure OvL and MFL in March 2015. Ovaries were hand-pollinated to produce immature and mature fruits, and three fruits from the sixth node of each plant were measured for fruit length (OvL and MFL) using digital callipers. The traits were measured according to the standards published by Yuan *et al.*^6^. All measurements were obtained on individual plant and averaged within each serial number. Subsequent data were analysed with analysis of variance and partial correlations using Microsoft Excel 2013.

### DNA isolation

Genomic DNA was extracted from young healthy leaves using the cetyltrimethyl ammonium bromide (CATB) method^36^. DNA concentration and quality were examined with the Qubit® 2.0 fluorometer (Invitrogen-Molecular Probes, Eugene, OR) and 1% agarose gel electrophoresis with a standard lambda DNA and a ND-1000 spectrophotometer (NanoDrop, Wilmington, DE, USA).

### QTL analysis

Using 148 F_2_ plants from CC3 × NC76, we previously constructed a saturated cucumber genetic map compromising 1800 SNPs. The F_3_ families used for fruit length data collection were derived from the same F_2_ population. In the present study, we re-genotyped the 1800 SNPs using Highmap^22^ software and corrected genotypes of some SNPs to generate a revised genetic map with the same number of SNPs. Details of genotype corrections and information of this SNP-based map are presented in Supplementary Table S1. The original SNP information is reported in Wei *et al.*^9^

QTL analysis was performed with R/qtl (http://www.rqtl.org/) and MapQTL®6 (https://www.kyazma.nl/index.php/mc.MapQTL/) using the revised F_2_ genetic map. QTL models adopted in R/qtl and MapQTL were CIM and MQM, respectively. Means of fruit length within each F_3_ family in spring 2013 and autumn 2014 were calculated and subjected to QTL analysis. The support intervals for map locations of QTL were calculated using a 1.5 likelihood-ratio statistic (LOD) drop interval. To declare the significance of QTL, LOD threshold was determined using a permutation test with 1000 repetitions (P = 0.05). The QTL was named according to its chromosome location and trait name. For example, *fl1.1* and *mfl3.1* refer to the first QTL for the length of immature and mature fruits on cucumber chromosomes 1 and 3, respectively.

### QTL-seq

Equal amounts of DNA were sampled from individuals with extreme long (4.0–6.0 cm) or short (1.3–2.0 cm) ovaries (15 DNA samples for each extreme trait) and bulked to generate the ‘Longest’ pool (L-pool) and ‘Shortest’ pool (S-pool). Pair-end sequencing libraries with a read length of 100 bp and insert sizes of approximately 500 bp were subjected to whole-genome resequencing with Illumina HiSeq 2500. Short reads obtained from both parents and two DNA-bulks were aligned against the cucumber genome sequence (the 9930 reference genome) to obtain the consensus sequence using BWA software^24^,^37^. Reads of L-pool and S-pool were separately aligned to CC3 and NC76 consensus sequence reads to call SNPs with SAM tools software^37^.

We calculated the SNP-index and Δ (SNP-index) to determine candidate fruit length-related QTLs^16^,^38^. The SNP-index refers to the proportion of reads harbouring a SNP when aligned to genome sequences of either parent. The average SNP-index of SNPs in a certain genomic interval was calculated using a sliding window analysis with 1 Mb window size and 10 kb increment. Δ (SNP-index) is the difference between SNP-index of L-pool and that of S-pool. Using CC3 as reference genome, SNP-index was 0 when all short reads contain genomic fragments from CC3, and SNP-index was 1 when all short reads contain genomic fragments from NC76. To ensure the accuracy of QTL detection, genome sequence of NC76 was also used as reference, and the SNP-index was similarly calculated. A SNP-index of 0.5 indicates equal contribution from both paternal genomes. Thus, most short reads are from both parents, except regions that significantly contribute to certain phenotypic variation. Δ (SNP-index) presents a strengthened result because it can eliminate background interference. The SNP-index graphs and corresponding Δ (SNP-index) graph were plotted. Statistical confidence intervals of Δ (SNP-index) was calculated under the null hypothesis of no QTLs followed the description by Takagi H. *et al.*^16^.

### Physical mapping and Gene Ontology (GO) annotation

Physical mapping of the genes homologous to fruit size onto the cucumber genome was performed by conducting BLASTN search of respective CDS sequences against the cucumber genome. Subsequently the genes were plotted onto the seven chromosomes according to their ascending order of physical position (bp), from the short arm telomere to the long arm telomere and ultimately the map was displayed using MapChart^39^.

We used Blast2GO^40^ to assign GO terms to cucumber genes. The GO term enrichment analysis was conducted for fruit size homologues in cucumber. The six homologous genes co-localized within QTL *FL3.2* was annotated using Cucurbit genomics database (http://www.icugi.org).

### Quantitative PCR (qPCR) analysis of candidate genes

Ovaries at 0, 2, 4, and 6 days before flowering from both parents were obtained and frozen in liquid nitrogen. Experiments were performed with two biological replicates (2 fruits from 2 plants) for each parent. Total RNA was isolated, and DNase I (Fermentas) digestion was performed for 30 min at 25 °C to remove DNA according to manufacturer’s instructions. cDNA was synthesized from 2 mg of total RNA using a cDNA Synthesis Kit (Fermentas). Quantitative real-time PCR was performed with a SYBR Premix Ex TaqTM Kit (TAKARA) in a Bio-Rad iQ1 real-time PCR system (Bio-Rad) as described by Li *et al.*^41^. The threshold cycle (Ct) value of each gene was investigated and normalized to the Ct value of *Cs-Actin*. To determine relative expression fold differences for each gene during different treatments, the 2^−△△Ct^ formula was applied. PCR primers were designed with Primer Premier 5.0 software (Premier Biosoft International) to avoid the conserved region. Details of the primer sequences are presented in Supplementary Table 5. Three replicates were used for the qRT-PCR. Analysis of relative mRNA expression data was performed using the Δ Ct method.

## Additional Information

**How to cite this article**: Wei, Q.-z. *et al.* Rapid identification of fruit length loci in cucumber (*Cucumis sativus* L.) using next-generation sequencing (NGS)-based QTL analysis. *Sci. Rep.* **6**, 27496; doi: 10.1038/srep27496 (2016).

## Supplementary Material

Supplementary Figures

Supplementary Table S1

Supplementary Table S2

Supplementary Table S3

Supplementary Table S4

## Figures and Tables

**Table 1:** Summary of QTLs detected for immature and mature fruit length using MapQTL and R/qtl in F3 families in spring 2013 and autumn 2014.

QTL	Chr	Data for QTL analysis	QTL package	1.5 LOD interval (CM)		Chromosome locations (Mb)		Peak location (Mb)	Interval(Mb)	LOD score	% Expl.	SNP NO.	Consistently QTL
				Left	Right	start	end						
*fl1.1*	1	2014, autumn	R/qtl	35.83	41.91	7.27	9.18	8.78	1.92	5.95	*	19	*FS1.1*, Weng *et al.*^2^
*mfl1.1*	1	2013, spring	R/qtl	61.31	70.50	13.30	16.46	13.51	3.16	11.73	*	8	*FS1.1*, Weng *et al.*^2^; *fl1.1*, Cheng *et al.*^28^; *mfl1.1*, Bo *et al.*^10^; *mfl1.2*, Wei *et al.*^9^
	1	2013, spring	MapQTL	64.35	74.70	13.47	17.43	16.48	3.97	6.23	17.34	12	
	1	2014, autumn	R/qtl	69.35	74.02	13.41	17.66	15.15	4.25	10.12	*	9	
	1	2014, autumn	MapQTL	71.99	73.35	16.48	16.84	16.52	0.36	5.94	20.08	4	
*fl3.1*	3	2013, spring	R/qtl	41.85	50.10	9.76	10.17	10.12	0.41	7.11	*	9	*FS3.1*, Weng *et al.*^2^
*fl3.2*	3	2014, autumn	MapQTL	118.18	139.49	22.71	28.20	26.37	5.50	12.81	36.76	23	*FS3.2*, Weng *et al.*^2^;*fl3.2*, Yuan *et al.* 2008;*mfl3.1*, Wei *et al.*^9^
	3	2013, spring	MapQTL	118.52	136.51	22.72	27.67	26.37	4.95	15.59	38.78	27	
	3	2013, spring	R/qtl	121.70	130.14	25.37	27.02	26.37	1.65	13.80	*	20	
	3	2014, autumn	R/qtl	121.70	130.14	25.37	27.02	25.83	1.65	15.69	*	20	
*mfl3.1*	3	2013, spring	R/qtl	29.28	38.40	7.57	9.03	8.09	1.45	6.36	*	13	*FS3.1*, Weng *et al.*^2^
*mfl3.2*	3	2013, spring	R/qtl	121.70	130.14	25.37	27.02	25.86	1.65	18.38	*	20	*FS3.2*, Weng *et al.*^2^;*fl3.2* Yuan *et al.* 2008;*mfl3.1*, Wei *et al.*^9^
	3	2013, spring	MapQTL	123.05	137.32	25.54	28.10	26.37	2.56	14.16	36.16	32	
	3	2014, autumn	MapQTL	135.70	142.67	27.51	29.98	26.37	2.48	10.05	30.87	15	
	3	2014, autumn	R/qtl	136.85	143.48	27.98	30.54	29.98	2.57	12.99	*	12	
*fl4.1*	4	2014, autumn	R/qtl	36.13	39.31	11.45	11.71	11.54	0.26	4.34	*	4	*FS4.1*, Weng *et al.*^2^;*fl4.1* Yuan *et al.* 2008;*mfl4.1*, Wang *et al.*^29^;*fl4.1*, Bo *et al.*^10^
*fl6.1*	6	2013, spring	MapQTL	31.78	41.24	11.11	15.50	14.13	4.39	6.43	17.76	26	*fl6.1*, Bo *et al.*^10^; *fl6.1*, Wei *et al.*^9^
	6	2014, autumn	R/qtl	33.47	36.17	11.64	14.13	12.72	2.49	4.76	*	22	
	6	2014, autumn	MapQTL	35.50	37.86	12.72	14.83	13.99	2.11	5.10	16.49	17	

**Table 2:** Summary of QTL sequencing data for each sample.

Name	Sample Clean Base (bp)	Clean Q20 (%)	Clean GC Content (%)	Sample coverage rate (%)	Map reads rate (%)	Uni hit reads rate (%)	Sequencing depth	Effective depth
L-pool	13324160800	94.26	38.1	91.34	61.98	54.43	65.73	42.69
S-pool	10859863600	93.84	37.99	92.28	61.03	52.54	53.58	34.22
CC3	5075004600	94.09	38.69	92.86	62.55	54.59	25.04	16.41
NC76	6472593400	94.01	38.22	93.31	62.93	54.47	31.93	21.04

**Table 3:** Summary of QTLs detected for ovary length with QTL-seq in RIL populations in spring 2015.

QTL	Cucumber Chr.	Chromosome locations (Mb)		Interval (Mb)	△SNP-index		Parent for QTL-seq analysis
		start	end		min	max	
*ovl1.1*	1	10.42	12.09	1.67	0.48	0.49	NC76
*ovl1.2*	1	16.92	18.83	1.91	0.48	0.51	CC3
	1	16.95	20.20	3.25	0.48	0.51	NC76
*ovl2.1*	2	10.73	12.76	2.03	0.52	0.57	CC3
	2	10.77	12.76	1.99	0.52	0.57	NC76
*ovl3.1*	3	11.12	16.45	5.48	0.71	0.80	NC76
	3	11.25	15.45	4.20	0.71	0.80	CC3
*ovl3.2*	3	25.97	30.55	4.58	0.70	0.74	NC76
*ovl6.1*	6	22.55	25.12	2.57	0.42	0.46	CC3
	6	22.57	24.75	2.18	0.44	0.50	NC76
