# Supplementary material for: Rapid identification of fruit length loci in cucumber (Cucumis sativus L.) using next-generation sequencing (NGS)-based QTL analysis
Source: Sci Rep. 2016 Jun 7;6:27496. doi: 10.1038/srep27496 (PMC4895147; doi:10.1038/srep27496)

Rapid identification of fruit length loci in cucumber (*Cucumis sativus* L.) using next-generation sequencing (NGS)-based QTL analysis

Qing-zhen Wei, Wen-yuan Fu, Yun-zhu Wang, Xiao-dong Qin, Jing Wang, Ji Li, Qun-feng Lou* and Jin-feng Chen*

Institution for all authors: State Key Laboratory of Crop Genetics and Germplasm Enhancement, College of Horticulture, Nanjing Agricultural University, Nanjing 210095, China

*Corresponding to Qun-feng Lou (qflou@njau.edu.cn) and Jinfeng Chen (jfchen@njau.edu.cn)

Submitting author: Qunfeng Lou

Postal address: College of Horticulture, Nanjing Agricultural University, Weigang Street No.1, Nanjing 210095, China.

Email addresses:

Qing-zhen Wei: 2013204012@njau.edu.cn

Wen-yuan Fu: 2014104070@njau.edu.cn

Yun-zhu Wang: 2012204011@njau.edu.cn

Xiao-dong Qin: 11109126@njau.edu.cn

Jing Wang: 2013104044@njau.edu.cn

Ji Li: liji1981@njau.edu.cn

Qun-feng Lou: qflou@njau.edu.cn

Jin-feng Chen: jfchen@njau.edu.cn

Supplementary Figure S1: Correlation of the genetic and physical positions of revised SNP-based genetic map. Axis of abscissa represents the genetic group, axis of ordinate represents the physical positions.

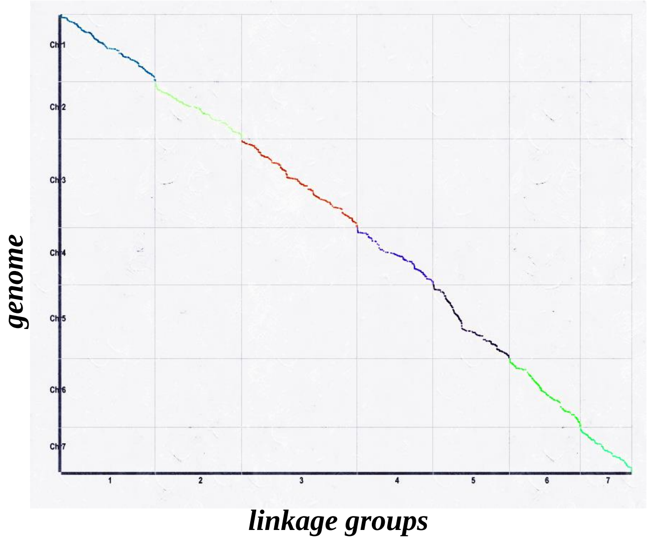

Supplementary Figure S2: QTL analysis of fruit length-related loci using MapQTL and R/qtl software packages in spring 2013 and autumn 2014.

QTL analysis using MapQTL in spring 2013

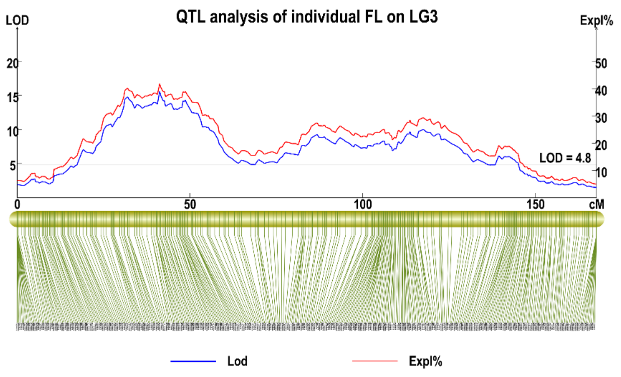

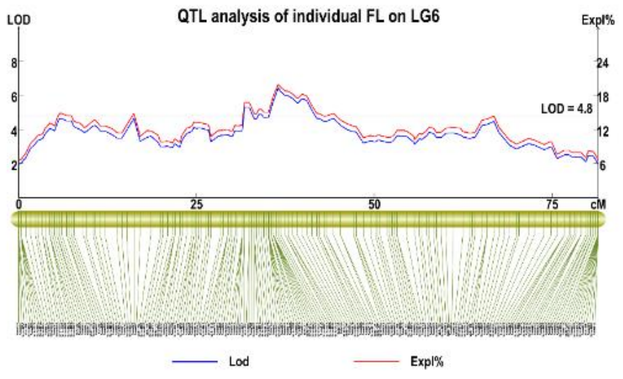

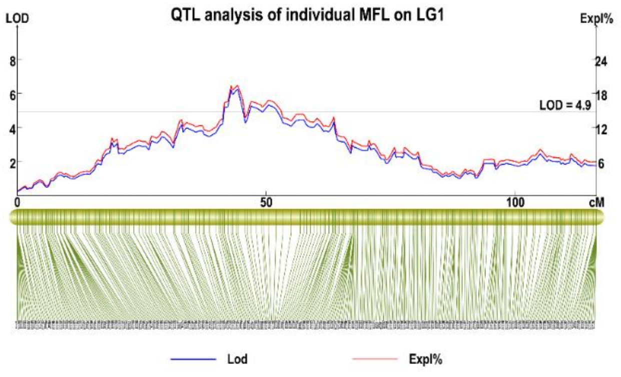

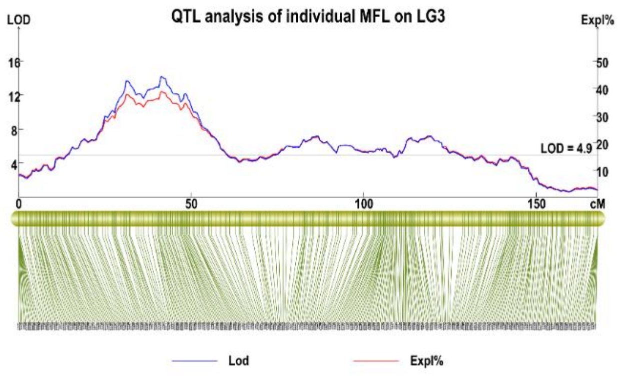

QTL analysis using MapQTL in autumn 2014

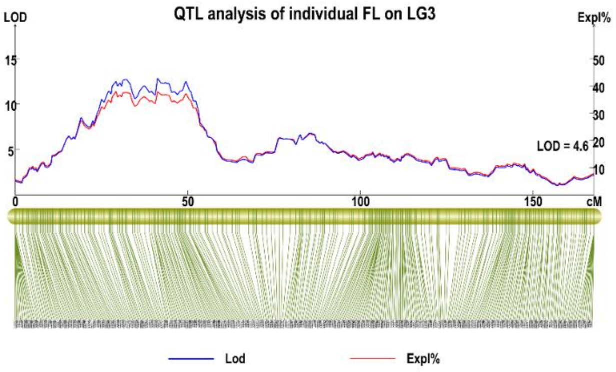

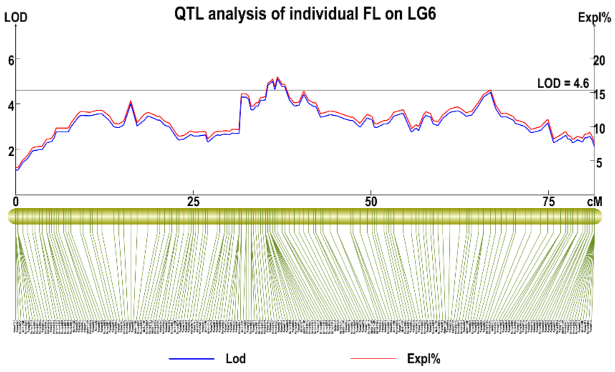

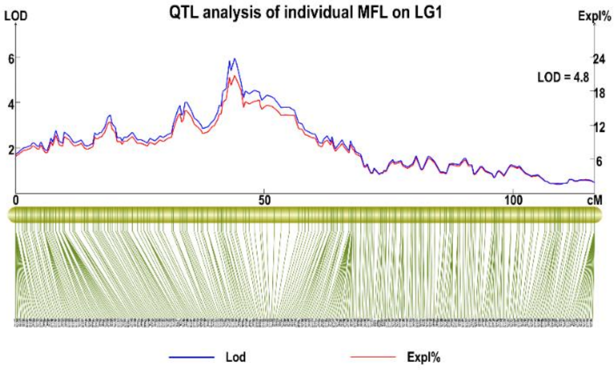

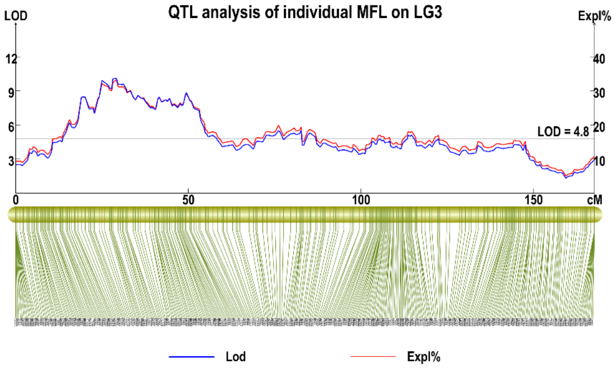

QTL analysis using R/qtl

QTL analysis of FL in spring 2013

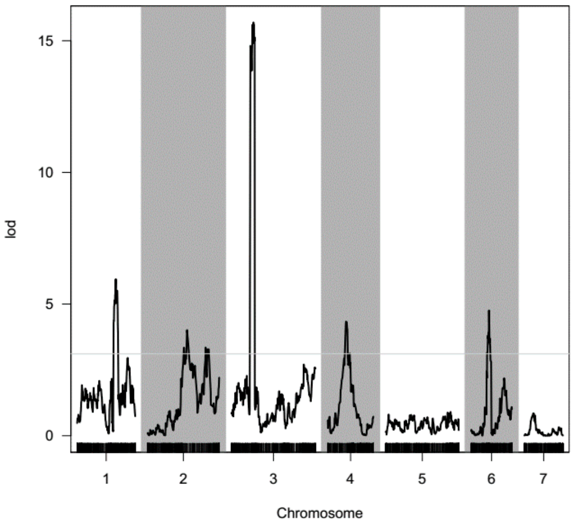

QTL analysis of MFL in spring 2013

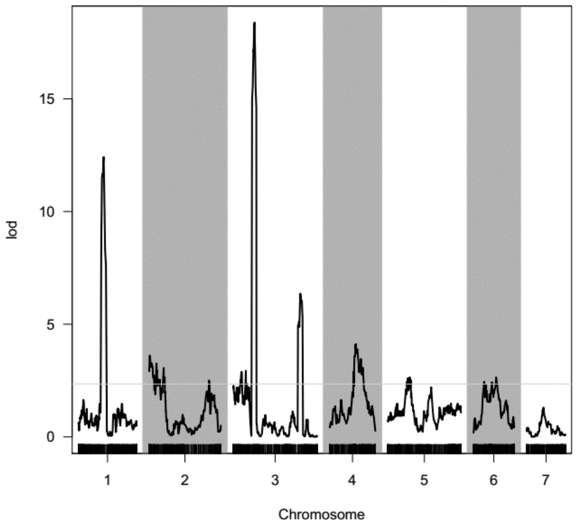

QTL analysis of FL in autumn 2014

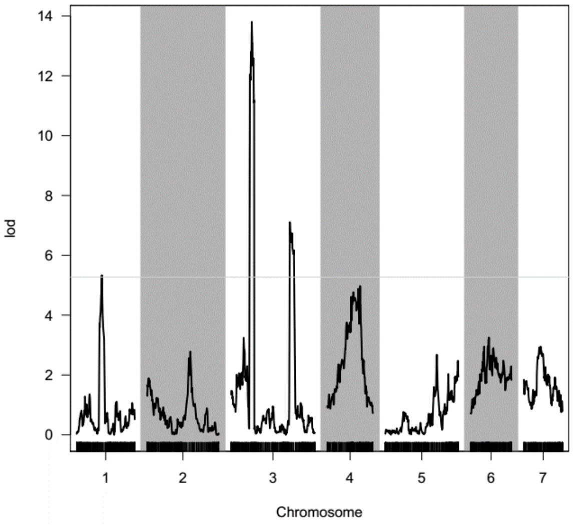

QTL analysis of MFL in autumn 2014

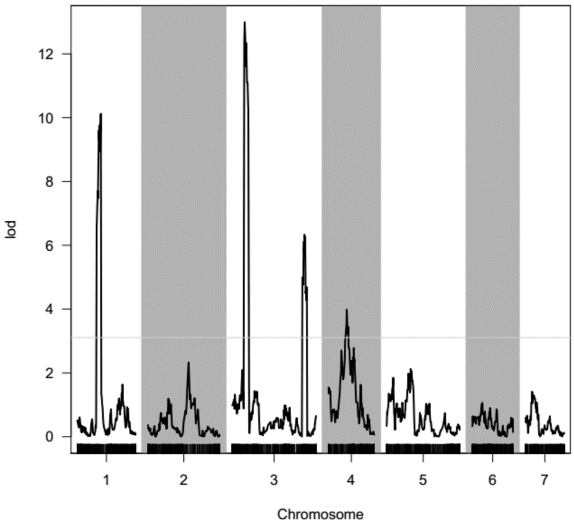

Supplementary Figure S3: Gene ontology (GO) analysis (P < 0.05) of the fruit size homologues in cucumber. GO terms belong to biological processes (GOBP), molecular functions (GOMF), and cellular components (GOCC) were shown in green, blue, and red, respectively.

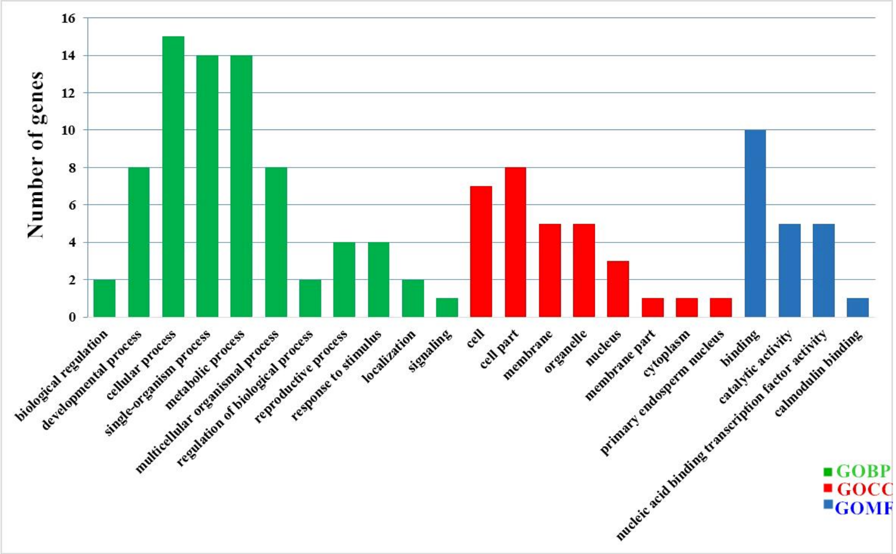

Supplement: Supplementary Figures [file srep27496-s1.pdf]
